# Physiologic Status Monitoring via the Gastrointestinal Tract

**DOI:** 10.1371/journal.pone.0141666

**Published:** 2015-11-18

**Authors:** G. Traverso, G. Ciccarelli, S. Schwartz, T. Hughes, T. Boettcher, R. Barman, R. Langer, A. Swiston

**Affiliations:** 1 Division of Gastroenterology, Massachusetts General Hospital, Harvard Medical School, Boston, MA, United States of America; 2 Department of Chemical Engineering and Koch Institute for Integrative Cancer Research, Massachusetts Institute of Technology, Cambridge, MA, United States of America; 3 Bioengineering Systems and Technologies, Massachusetts Institute of Technology Lincoln Laboratory, Lexington, MA, 02420, United States of America; Radboud University Nijmegen, NETHERLANDS

## Abstract

Reliable, real-time heart and respiratory rates are key vital signs used in evaluating the physiological status in many clinical and non-clinical settings. Measuring these vital signs generally requires superficial attachment of physically or logistically obtrusive sensors to subjects that may result in skin irritation or adversely influence subject performance. Given the broad acceptance of ingestible electronics, we developed an approach that enables vital sign monitoring internally from the gastrointestinal tract. Here we report initial proof-of-concept large animal (porcine) experiments and a robust processing algorithm that demonstrates the feasibility of this approach. Implementing vital sign monitoring as a stand-alone technology or in conjunction with other ingestible devices has the capacity to significantly aid telemedicine, optimize performance monitoring of athletes, military service members, and first-responders, as well as provide a facile method for rapid clinical evaluation and triage.

## Introduction

Heart rate (HR) and respiratory rate (RR) are essential vital signs for evaluating the physiologic status of children and adults in clinical and non-clinical settings. These two vital signs constitute the initial measurements in acutely ill patients and provide the basis for clinical severity stratification [1, 2] as well as markers of response to life-saving cardiopulmonary resuscitation [3]. Additionally, HR and RR serve as non-diagnostic indicators of performance status in service members [4, 5] and in performance athletes.

There are numerous methods for monitoring HR and RR, but most require the attachment of superficial sensors to the body. HR can be monitored using electrical methods such as electrocardiogram (ECG), optical methods such as photoplethysmography (PPG, pulse oximetry), or mechanical methods such as ballistocardiography. RR can be monitored directly using trans-thoracic plethysmography and expired gas analysis approaches, or indirectly using advanced processing methods applied to PPG [6]. All of these methods have some limitations, as they may cause patient discomfort by being obtrusive or irritating the skin[7, 8], and many methods cannot be used reliably in high physical activity contexts where motion may corrupt the signal. Furthermore, some key vital signs, namely core temperature, must be measured internally, and thus an ingestible device can provide the best signal quality. Over the last decade, ingestible medical devices have gained broad acceptance; for example, ingestible devices can measure temperature [9, 10], and video capsule endoscopy is widely used for diagnosis of gastrointestinal (GI) pathology [11]. We hypothesized that vital sign monitoring from within the GI tract would be a safe and effective alternative to existing superficial clinical monitoring systems, while overcoming some of their limitations. Moreover, we selected miniature components in our design to ensure that the ultimate size of ingestible PSM devices is even smaller than video capsule endoscopes while maintaining other safety standards.

We present initial proof-of-concept experiments in a porcine model to show that HR and RR can be measured simultaneously and with high fidelity from within the GI tract using a single acoustic waveform. Using an endoscopically-guided miniature electret microphone, we measured acoustic data along the GI tract from the mouth to the colon. We evaluated the impact device contact with GI tissue and previously ingested food had on acoustic data quality. We then developed a robust signal processing algorithm to analyze these raw waveforms. Our results support that an ingestible, ultra-miniature acoustic monitoring device could accurately measure vital signs. This technology is likely to be adaptable to a wide range of clinical and non-clinical uses.

## Results

### Data Collection and Signal Processing

**Fig 1:**
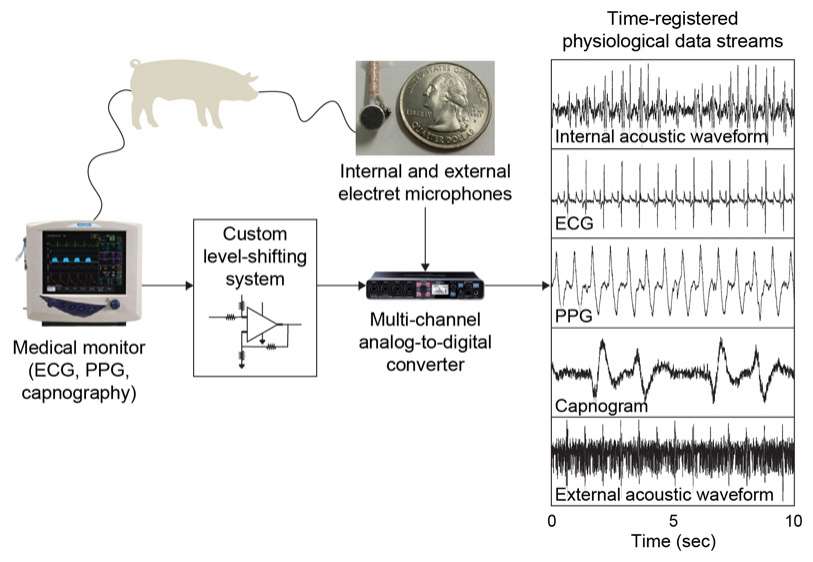
Schematic of the experimental setup and representative dataset. The animal model is anesthetized and attached to a medical monitoring system that measures ECG, capnography, and photoplethysmography (PPG) waveforms simultaneously. We built a custom voltage level-shifting circuit for each waveform, which outputs to a commercial A/D converter. Two electret microphones, one controlled internally by the endoscope, the other attached superficially on the pig’s chest just above the heart, are also sending data to the A/D converter. The final result is perfectly time-registered data streams for heart and lung function as well as the acoustic waveforms. Example concurrent physiological data measurements were taken from the proximal third of a porcine stomach including the acoustic waveform from our internal electret microphone, ECG, PPG (which indicates systemic oxygen perfusion levels and heart rate), capnography from expired CO_2_ content, and the acoustic waveform from the external microphone positioned above the heart. (Note that the raw PPG data from the SurgiVet system seems to be inverted, and the raw capnography data appears to be the flow *rate* of CO_2_, thus giving a first derivative of the more familiar capnogram waveform.).

We performed physiological monitoring experiments in six sedated Yorkshire pigs using an endoscopically-guided electret microphone to collect acoustic waveforms along the GI tract. We concurrently recorded physiological waveforms using a standard veterinary vital signs monitor, including external 3-lead ECG, PPG, capnography (via expired breath CO_2_ analysis), and a superficial microphone positioned directly above the heart. A total of ~407 minutes each of HR and RR data were collected from all segments of GI tract over the course of four experimental days in six Yorkshire pigs. Specifically, 80.3, 66.7, 149.7, 54.3, and 60.3 minutes of raw audio data were collected from the oral cavity, esophagus, stomach, proximal duodenum and rectum respectively. A schematic of our experimental setup and a representative dataset is shown in Fig 1.

**Fig 2:**
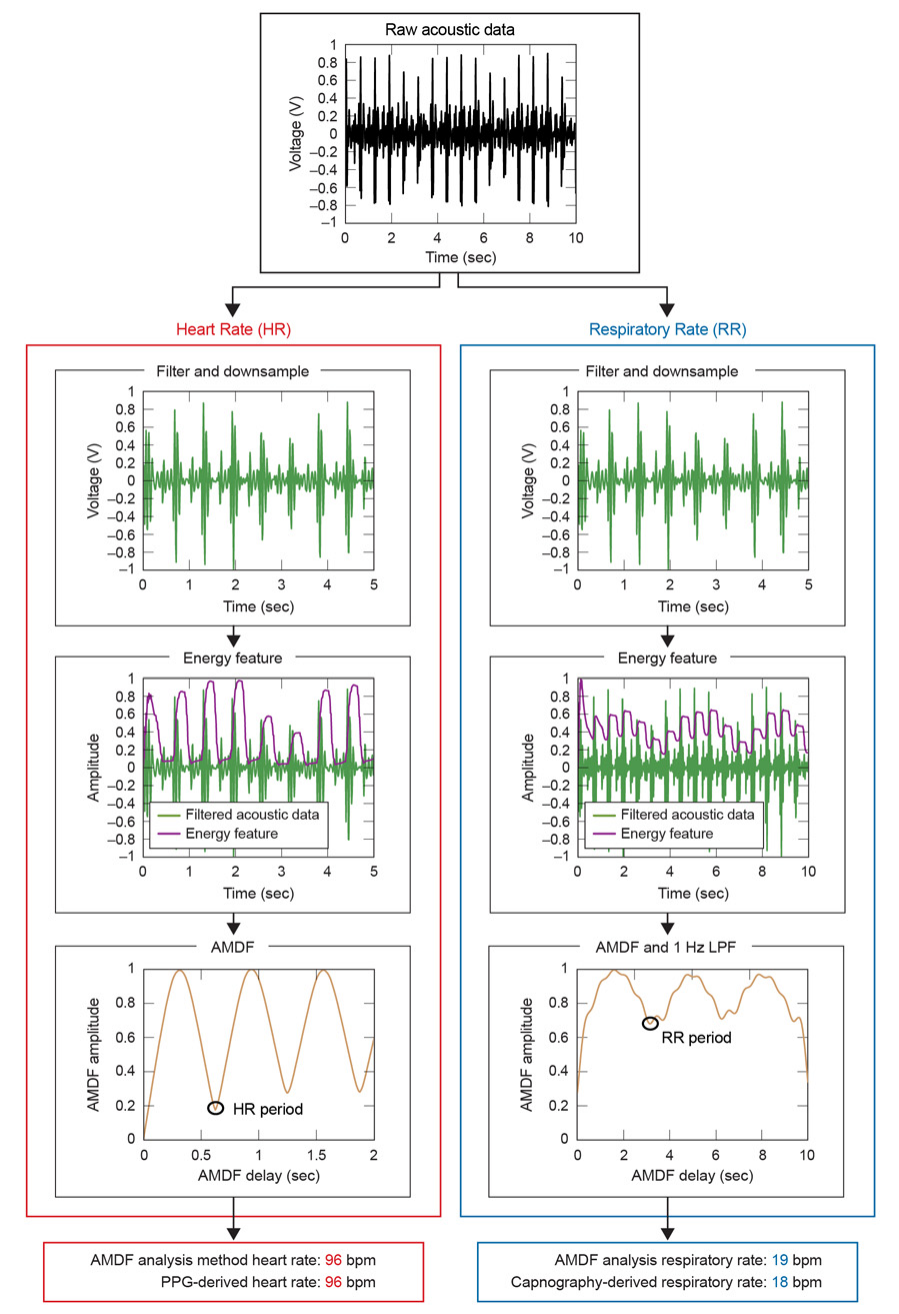
Schematic processing flow chart for HR and RR estimation from internal microphone data. The signal is copied into a HR track and RR track and then analog filtered and down-sampled. A sliding window computes an energy feature (see Methods) that is input to the average magnitude difference function (AMDF). The RR is further low pass filtered with a 1 second Hamming window. The first valley of the AMDF is the estimated vital sign.

**Fig 3:**
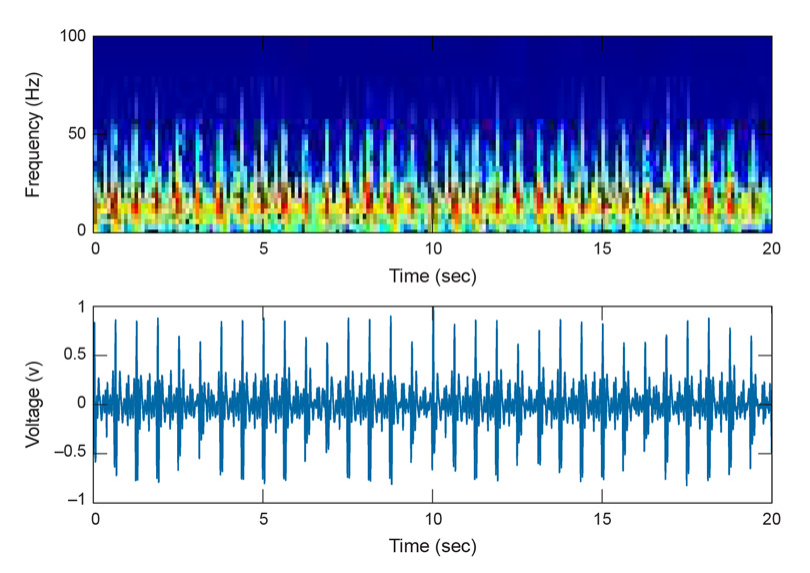
Example spectrogram and corresponding time course data of the internal acoustic signal measured in the proximal third of the stomach. A majority of the signal energy is <50Hz.

Raw waveforms were processed using a phonocardiogram HR estimation algorithm [12] modified to enable simultaneous extraction of HR and RR from a single raw waveform and with processing steps amenable to implementation on a microcontroller (such as a Texas Instruments MSP430). The raw waveform is split and copied into a HR and RR track, and each is processed with parameters characteristic of each signal [13–15] (see Fig 2). The first processing stage consists of an emulated analog front end, namely resistor-capacitor (RC) based bandpass filters. A 10–40 Hz anti-aliasing filter is applied to capture the majority of signal energy yet avoid significant aliasing from downsampling (see spectrogram in Fig 3). The second and third stages increase the signal-to-noise ratio (SNR) by successively computing the signal’s energy and its average magnitude difference function (AMDF). The final stage uses a robust valley-detection algorithm for estimating HR and the RR from the AMDF. All processing in this study was performed on non-overlapping 20s data frames during which a single average HR and RR is reported. A 20s frame duration was chosen as a compromise between reduced latency and a sufficient window length to encompass more than one breath. There were 1228 and 1219 total HR and RR frames, respectively. Additional signal processing details may be found in the Methods section.

**Fig 4:**
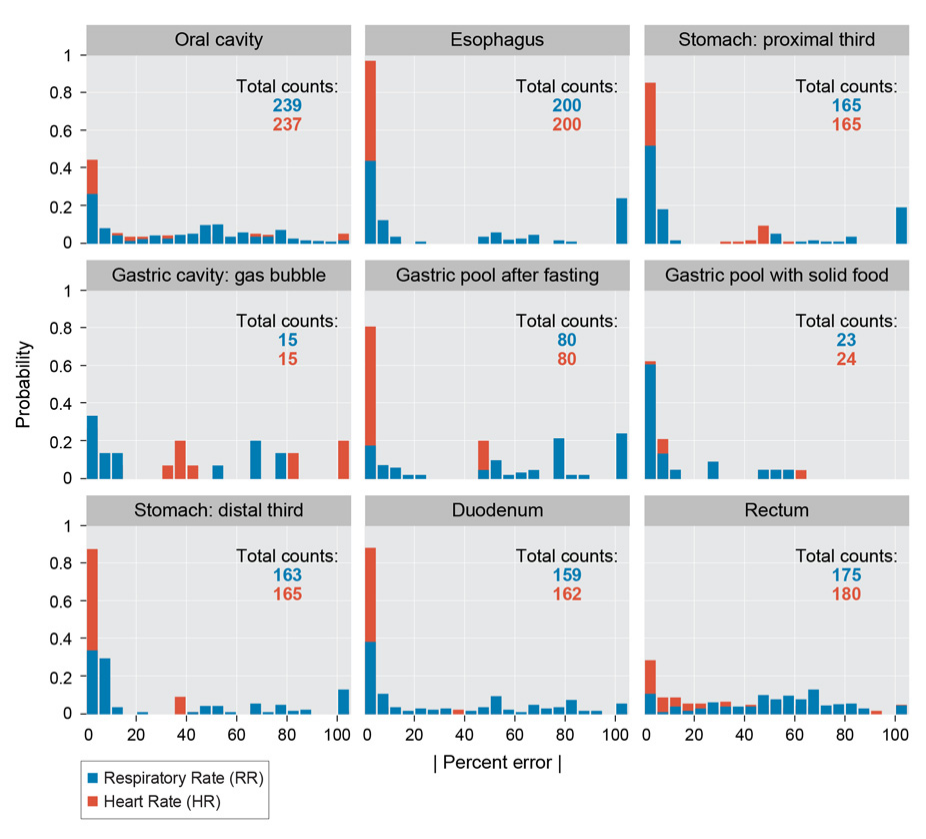
HR and RR estimation performance histograms for all data collected as a function of anatomical location. The x-axis represents the absolute value of the percent error in a given 20s frame; the y-axis is the number of such frames normalized by the “Total Counts” (for each location and vital sign) used to build the histogram. The AMDF valley-finding algorithm can trigger upon higher-order harmonics of the fundamental period, on noise, or on the incompletely removed heart rate period, giving percent errors concentrated at 50% and 100%; these errors can be easily addressed with more sophisticated AMDF algorithms or running median filters (see Discussion).

HR concordance with external PPG is very strong in the esophagus, stomach (including with and without food and tissue contact), and duodenum: the HR is detected within 5 bpm 97%, 95%, 98% of the time, respectively. The RR is detected within 5 breaths per minute 84%, 82%, 88% of the time in these locations, though many errors in these locations are due to the AMDF algorithm’s valley-finding which very predictably doubles the respiratory rate (see Discussion below). Resting respiratory and heart rate in swine ranges are noted between 32–58 breaths/min and 70–120 beats/min respectively[16]. Our 10%:90% quantiles for respiratory and heart rate were 15:43 and 57:107 bpm. While we did measure an acoustic waveform in the mouth and colon, agreement with standard vital sign monitoring was poor (see detailed analysis in Fig 4 and Discussion below). We measured ambient operating room noise during data collection which averaged ~70dB, and peaked at ~80dB.

### Vital sign monitoring in heterogeneous GI environments

**Fig 5:**
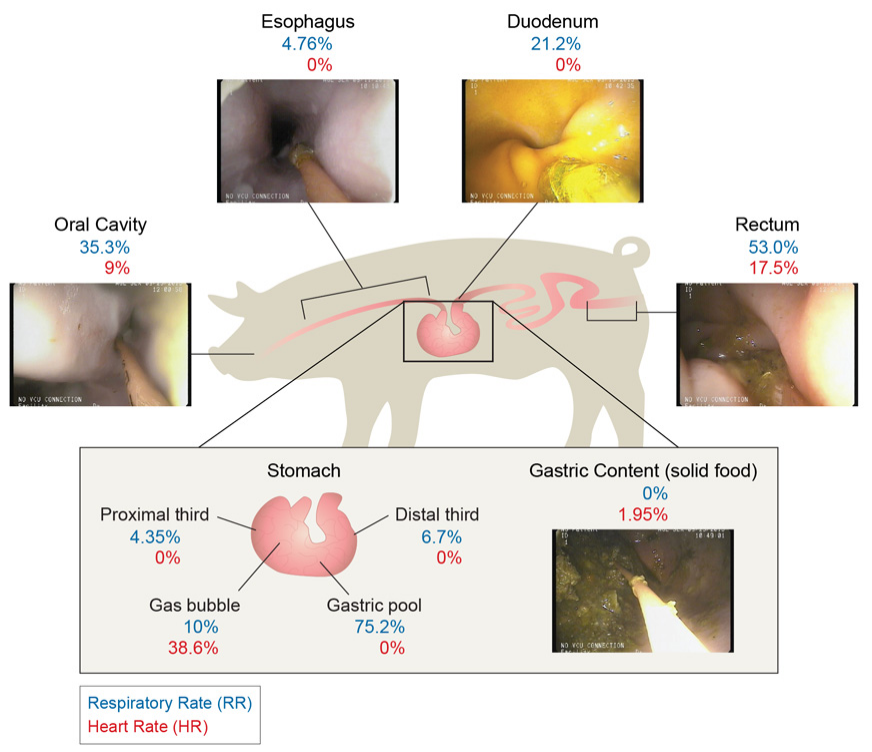
Vital sign algorithm performance as a function of anatomical site. Median absolute percentage error between PPG and capnography derived HR and RR and acoustically derived HR and RR via our average magnitude difference function (AMDF)-based analysis is reported at each measurement site.

An ingestible monitoring device must be able to function under a variety of common GI environmental conditions, including fasted and fed states and device contact with tissue. To demonstrate our system’s applicability to these heterogeneous GI environments, we measured waveforms in the gastric content (either solid, liquid, or in a gas bubble, for a total of 39.7 minutes of data collected from the six animals), and in contact with the gastric wall (approximately 110 minutes of data collected from the six animals, both in the proximal and distal thirds of the stomach). All these areas demonstrated HR and RR values in good agreement with external vital sign monitor values. The HR median absolute percent error was noted to be 0% in the proximal third, gastric pool, and distal third. For RR, the median percent error was 4.3%, 10%, and 6.7% in the proximal third, gas bubble, and distal third. Additional median percent errors are noted in Fig 5.

## Discussion

Physiological status monitoring is central to the clinical evaluation of patients and increasingly used in non-clinical settings for safety (for example, military service members and first-responders) and performance monitoring (such as professional athletes). Though significant development has focused on low-profile external vital sign monitoring systems, extended monitoring in *non-wearable* formats has seen little development. Wearable systems can be associated with skin irritation from allergic responses or repetitive abrasion during extended use in high physical activity settings, as well as from constriction, and all are subject to the logistical burdens of user compliance. Furthermore, wearable systems are not always capable of directly measuring some key physiological variables externally, namely core temperature.

To address these limitations, we have established a new technique for physiologic status monitoring using technologies and methods compatible with ingestion. Our method is capable of sensing primary vital signs (HR and RR) in a heterogeneous set of environments in the GI tract using a single sensing modality. We have demonstrated measuring HR and RR using this technique *in vivo* in a large animal model at various locations along the GI tract. Signal fidelity was maintained with and without microphone tissue contact, as well as within solid and liquid food material. Our signal processing algorithm robustly detected HR and RR in excellent agreement with measurement from standard external vital signs monitors (PPG and capnography) in the majority of the GI tract.

Ingestible devices have a number of advantages over wearable systems. The ergonomic profile of an ingestible-sized device is minimal; unless a patient has a specific contra-indication for such a device (bowel obstruction, etc.), the user will remain asymptomatic for the duration of monitoring. Such small devices will be similar in size to a bolus of food or pharmaceutical capsule (the largest commonly used size, 000, is ~2.5cm long x 1cm diameter) and will remain free-floating in the GI tract; with an appropriate material choice for the device capsule, this will minimize any abrasive irritation of the GI mucosa. There is also the possibility of implementing this sensor concept as a dissolvable electronic device, thereby eliminating contra-indications. Furthermore, ingestible electronic systems offer the possibility for *in vivo*, yet *non-implantable* medical devices able to monitor difficult-to-measure variables (such as core temperature) simultaneously with HR and RR. Burdens associated with implantable devices, namely the need for a surgeon to introduce the device and potential negative health outcomes (infection, fibrosis), have prevented their wide adoption, and which ingestible devices overcome.

Our signal processing algorithm produced accurate HR estimates for the majority of the GI tract. Overall HR performance is very strong in the esophagus, stomach, and duodenum: concordance with external PPG is detected within 5 bpm 97%, 95%, 98% of the time, respectively. While we did measure an acoustic waveform in the mouth and colon, agreement with standard vital sign monitoring was poor. Because of the excellent performance of the sensor and algorithm proximal to the heart and lungs, we suspect these sites were too distant from the heart and lungs for the sensitivity of the particular microphone chosen (-45dB ±4dB). Higher sensitivity electret or MEMS microphones may be chosen, but must also be small enough for ingestion and have sufficient frequency sensitivity (from 10 to 40 Hz for both HR and RR).

Percent error histograms (Fig 4) show similar empirical distributions among anatomical locations when there are sufficient samples. Namely, measurement error is concentrated near 0%, 50%, and 100%. This consistent and repeatable distribution results from the AMDF valley-finding algorithm triggering on incorrect valleys. With no noise, the first AMDF function valley represents the fundamental period of the heart rate or breathing rate; however, there are additional valleys at multiples of this fundamental period due to the periodicity of the signal, small valleys due to incomplete removal of heart rate modulation in the respiratory track, and spurious smaller valleys due to noise. For example, a doubled pitch period yields errors of 50%. Any detections on spurious valleys before the first true valley result in over estimations of the parameter of interest; if these estimates occur at less than half the true period, the error value becomes > 100%, which are clustered together in Fig 4. The harmonic nature of the AMDF function and its corresponding error modes are a recognized failing of parameter estimation from a periodic waveform such as the AMDF. Potential improvements in future work include implementing more robust period estimation methods such as Extended AMDF [17], normalized autocorrelation, Comb transformation or cepstral estimation algorithms with a period tracker (such as a Kalman filter), or a combination of such methods.

To statistically measure our algorithm’s performance, we performed an equivalency test between HR and RR derived from our acoustic approach and the gold-standard value based upon the median difference between matched pairs. We similarly performed a test for correlation significance between acoustic estimates and gold-standard values. (See Methods for details on both.) For HR, the median error difference is equivalent to 0 (within +/- 5bpm at the 95% confidence level) for the esophagus, proximal third of stomach, distal third of stomach, gastric pool, stomach with food, and duodenum. Furthermore, our acoustic estimates are significantly positively correlated (at the 95% level) for each of these locations except the stomach with food and duodenum. Median significant correlations for HR are 1.0, 0.53, 0.79, and 0.61 for the esophagus, proximal third of stomach, distal third of stomach, and gastric pool, respectively.

For RR, the median error difference is equivalent to 0 (within +/- 5bpm at the 95% confidence level) for the esophagus, proximal stomach, and stomach with food, but we were unable to demonstrate significant correlation. As mentioned above, the full histograms (see Fig 4) imply particular susceptibility of our algorithm to pitch period doubling and low signal quality which can be mitigated with filtering above the 20s frame level as well as implementing signal-to-noise measures on the raw acoustic data and AMDF output. To assess algorithm output performance improvements if these issues were addressed, we removed respiration rate pairs with absolute percent errors greater than 35% (that is, data likely to be excluded using filtering and signal-to-noise measures) and performed our equivalency and correlation tests again. With this new limited dataset, all locations have a median difference of 0 and have significantly correlated acoustic and gold standard estimates (median correlations of at least 0.90 for mouth, esophagus, proximal and distal third of stomach, gastric pool, duodenum, and rectum, and 0.59 for stomach with food).

One considerable limitation to our proposed system is that GI transit time varies considerably among healthy individuals [18]. Our initial data suggests that our chosen components and processing algorithm yields best results in the upper GI tract (from the esophagus to the small intestine), and for some individuals, this residence time may be 12h or even shorter. However, this duration of monitoring is similar with many other ambulatory physiological monitoring systems, and this one of the same limitations of the existing “gold standard” ingestible core temperature monitoring solution (the VitalSense™ capsule system [9]). Another potential limitation is the effect of ambient noise pollution on acoustic signal fidelity. Reassuringly, data collected here appeared robust in spite of room noise contributions ranging from 70 to 80 dB. Noise pollution, either from ambient or internal sources, may also be addressed using more robust signal processing algorithms, such as match filtering for known physiological acoustic signatures. Further experimentation in various simulated and environmental contexts is required to fully characterize signal fidelity using this technology as well as inter-individual variability to inform adaptive on-board algorithms of future devices.

Extended vital sign monitoring via ingestible devices could be applied in emergency triage settings in the field, for post-operative patients, outpatient telemedicine monitoring, and performance measurements (such as in professional athletes and military service members). In this study we measured respiratory and heart rates ranging from 6–56 breaths/min and 48–128 beats/min with high correlation to standard monitoring devices. Further testing to the extremes encountered in active (i.e., exercising) or pathophysiologic states will be required to fully delineate the limitations of this technology for monitoring, but are the focus of current hardware and algorithm development efforts. Continuous auscultation, when coupled with more advanced signal processing algorithms focusing on anomaly detection or machine learning-based classifiers, could provide advanced diagnostic tools for pulmonary (chronic obstructive pulmonary disease, asthma, etc.) or cardiac (arrhythmias, stenosis, etc.) pathologies. Future vital sign device designs may even include coupling with *in situ* treatment delivery devices to provide remote, automated systems for rapid diagnosis and treatment of high-risk patients.

## Methods

### Porcine Model Vital Sign Monitoring Experiments

In vivo porcine studies were performed in 6 Yorkshire pigs weighing between 50 and 65kg. All animals were female and between 6–7 months of age. For evaluations free of food material, the pigs received a liquid diet for 48 hours prior to the procedure. Otherwise animals were fasted overnight. On the day of the procedure, the morning feed was held and the animal was sedated and intubated. Following induction of anesthesia with intramuscular injection of Telazol (tiletamine/zolazepam) 5mg/kg, xylazine 2mg/kg, and atropine 0.05 mg/kg, the pigs were intubated and maintained on isoflurane 1–3%. An endoscope guiding a miniature electret microphone (PUI Audio, part # POM-2245L-C10-R) was introduced in the esophagus and recordings taken from the mouth, esophagus, stomach, and duodenum with and without tissue contact. Additionally an enema was administered and recordings were taken from the colon. All procedures were conducted in accordance with protocols approved by the Massachusetts Institute of Technology Committee on Animal Care Protocol #0113-009-16. After data collection, all animals were returned to the animal colony and used in other experimental protocols.

Raw acoustic data was sampled at 44.1 kHz. We built a custom level-shifting device (shifting from 0-5V to 0–1.6V) capable of interfacing with existing medical monitoring equipment attached to the animal (Surgivet Advisor™, Smiths Medical) and with a multi-channel A/D converter (Roland Octa-Capture™) capable of handling all 5 input streams (ECG, PPG, capnography, internal and external electret microphones) and outputting via a USB connection to a laptop running Audacity audio collection software.

### Signal Processing Algorithm

Our algorithm was specifically designed for implementation in a low size, weight, and power device. First, the 44.1 kHz signal is segmented into 20s segments and copied into two parallel processing tracks; one track ultimately estimates HR, and the other RR. All acoustic data is highpass filtered with a 100dB attenuation Chebyshev Type II filter with a transition band from 5-10Hz to eliminate motion artifacts caused by mechanical compressions rather than acoustic vibrations. This filter is digitally implemented and would not necessarily be part of the final design. The signal in both HR and RR tracks is filtered with an emulated analog RC bandpass filter constructed from a low pass filter with a 3 dB cutoff frequency at 40 Hz and a high pass filter with a 3 dB cutoff frequency at 10 Hz. Finally a 60Hz comb filter is applied to reduce line noise and its harmonics. Both signals are then decimated by a factor of 450 to a sampling rate of 98 Hz to emulate minimal data collection, storage, and processing capabilities.

The analog filtered signal, *x*[*n*], is normalized to a maximum amplitude of -1 or 1 and is denoted *x*[*n*]_*norm*_. Then, a sliding window over the 20 second frame computes the energy, *E*[*n*], according to Eq 1 below. The HR track uses a window of *N*_*window*_ = 25 samples or approximately 0.25 seconds, and the RR track uses a window of *N*_*window*_ = 98 samples or 1 second.

$$\begin{array}{l} \text{If}\;1\leq n<N_{window}\\ \;E\left[n\right]=\frac{1}{n}\cdot \sum_{k=1}^{n}{\left(\;x_{norm}\left[k\right]\right)}^{2}\\ \text{else if}\;N_{window}\leq n\\ \;E\left[n\right]=\frac{1}{N_{window}}\cdot \sum_{k=1}^{N_{window}}{\left(\;x_{norm}\left[n-N_{window}+k\right]\right)}^{2} \end{array}$$ Eq 1. Energy feature calculation

The average magnitude difference function (AMDF) computes a waveform *D*[*n*] similar to the output from an autocorrelation operation, but without using any multiplications [19] (again, chosen in anticipation of implementing in ultra-low power and size hardware). The AMDF slides the signal over itself and computes the average difference between the overlapped segments (Eq 2). When the two segments are similar, the AMDF outputs a low value, and when they are dissimilar, the AMDF outputs a high value. The AMDF increases the signal to noise ratio by exploiting the periodicity of the HR and the RR waveforms over the frame. *N*_*E*_ is the number of samples in the frame of the energy signal (*N*_*E*_ = 1960, corresponding to 20 seconds). d varies from 0 to 196 (2 seconds) or 0 to 980 (10 seconds) for HR or RR estimation, respectively, because normal cardiac and respiration cycles have periods less than these upper bounds.

$$\begin{array}{l} N=N_{E}-d_{max}\\ D[d+1]=\frac{1}{N}\cdot \sum_{k=1}^{N}|E[k]-E[k+d]|\\ \;\text{where}\;d=0,1,2,..,d_{max}. \end{array}$$ Eq 2. Average magnitude difference function

For the RR track only, the AMDF output is further low pass filtered with a 1 second hamming window. This reduces the likelihood that high frequency heart rate modulation will be detected instead of the low frequency respiratory modulation. The final stage in each track is the HR and RR estimation from the AMDF function. Vital sign estimation reduces to a valley-detection-in-noise problem because the true HR or RR period will be the first significant dip at which the AMDF becomes close to zero (though not counting the AMDF value at zero lag, as this has an AMDF value of exactly zero since the two segments being subtracted are identical.) As the segments slide over each, the heart beat or breath that was well aligned in each segment becomes misaligned, and the AMDF value increases. However, eventually the segments will have slid far enough apart that the original heart beat or breath will overlap with the second heart beat or breath in the frame. Because of the consistency in heart rate and breath morphology as an output from the energy stage, the AMDF function will compute a small, non-zero value before increasing once again. If several heart beats are within a frame, the AMDF function will appear to have a ripple or sinusoidal pattern as the first heart beat overlaps each of the successive heart beats. The ripple period is the period of the vital sign to be estimated.

### Data Analysis & Statistical Measures

We compared the results from the signal processing algorithm above with the “gold-standard” PPG and capnography signals to determine algorithm performance. Data from these gold-standard methods was resampled to 98 Hz using MATLAB’s (MathWorks, Natick MA) resample function, which applies an anti-aliasing filter, and then processed through the AMDF and valley detection stages as described earlier. Only HR values between 40 and 200 beats per minute and RR values below 60 breaths per minute were accepted as physiologically reasonable. In the rare event (10/1229 frames for RR and 6/1234 frames for HR) that the valley finding function did not return an estimate from the acoustic data but the gold standard estimate was valid, the frame was excluded from analysis. For each 20s segment, an absolute percent error is calculated, defined as

$$Absolute\;Percent\;Error\;=\;100\;\times \left|\frac{Rate_{algorithm}-Rate_{gold-standard}}{Rate_{gold-standard}}\right|$$

where *Rate*_*algorithm*_ is the result of the method above, and *Rate*_*gold-standard*_ is the rate derived from PPG or capnography for HR and RR, respectively. Histograms of these values, normalized by the total counts for each anatomic site, are reported in Fig 4 and the median presented in Fig 5.

We used two methods to statistically determine output similarity between the gold-standard methods (PPG and capnography) and our acoustic approach. The first was a median difference between matched pairs for repeated samples equivalency test, which checks for bias while still being robust to outlier failures from period doubling; the second was a test for correlation significance, which quantitatively assesses whether our acoustic based estimates track the corresponding gold-standard vital signs.

For the equivalency test, the null hypothesis is that a non-tolerable difference exists between the acoustic and gold standard estimates, and the alternative hypothesis is that the median difference is equivalent to 0 within the tolerance limits (at the 95% confidence level). We chose a non-tolerable difference of +/- 5 bpm for both the HR and RR median differences. We performed a bootstrap procedure for the equivalency test given that our data is non-normal and has few independent samples (at most 6 independent pigs for a given anatomical location), though we have repeated samples for each pig at a given location. For each location, we randomly sampled one measurement pair (acoustic estimate and gold-standard value) from each pig that had data for that location. We computed the difference for the pair and took the median of the differences for all the pigs to create one bootstrapped datum. We repeated this procedure 10000 times to create a distribution of median difference errors, and then computed the 5 percentile and 95 percentile points. If both the 5 percentile and 95 percentile points lie within the tolerable difference region of +/- 5 bpm, we reject the null hypothesis (that the median is significantly different than 0 within the tolerance bounds) in favor of the alternative. Note that 5 and 95 percentile points are used for a 95% confidence level instead of 2.5 and 97.5 percentiles because both ends of the confidence interval must be within the tolerance bounds to reject the null hypothesis.

A correlation test was also performed with a similar bootstrapped approach. For each location, one measurement pair was taken for each pig. The Pearson correlation among pairs was computed to create one bootstrapped datum. We repeated this procedure 10000 times to create a distribution of correlations, and then computed the 2.5 and 97.5 percentile points. If the 2.5 to 97.5 percentile range did not include 0, then we reject the null hypothesis (that our acoustic measurements are uncorrelated with the gold standard) in favor of the alternative that data are correlated at the 95% confidence level.

In the case of only one pig being available for the stomach with food condition, the samples were treated as independent and half the samples were randomly picked to create a bootstrap datum. In one case, the gold-standard vital sign was constant, and consequently, we can only report on median difference error and not report a correlation value.
